# Apology

**Published:** 1855-01

**Authors:** 


WE regret the delay which has attended the appearance of our present number, but it was unavoidable. The lecture of Prof. MILLER on diseases of the os uteri, which we have the pleasure of laying before our readers, will be followed by two others on the same subject, and, we are sure, will be read with deep interest. No practitioner in our country has had greater experience in the treatment of such affections than Prof. MILLER, and his opinions on the subject carry with them great authority.

We have the promise of a series of lectures, by Prof. FLINT, on diseases of the skin, which, we believe, will give attractiveness to our journal. Their publication will be commenced as soon as the series by Prof. MILLER is completed.

The review of the treatise on foreign bodies in the air-passages, by Prof. GROSS, which we expected to insert in our present issue, has been crowded out by other matter, but will appear in our next number.

